# A Rare Case of Metastatic Malignant Melanoma to the Colon from an Unknown Primary

**DOI:** 10.1155/2014/312902

**Published:** 2014-06-30

**Authors:** Preethi Reddy, Courtney Walker, Bianca Afonso

**Affiliations:** ^1^Department of Medicine, University of Arizona, Tucson, AZ 85721, USA; ^2^Department of Medicine, University of Arizona Medical Center, 6th Floor, Room 6336, 1501 N. Campbell Avenue, Tucson, AZ 85719, USA; ^3^Division of Gastroenterology, Department of Medicine, University of Arizona, Tucson, AZ 85721, USA

## Abstract

Metastatic melanoma from an unknown primary (MUP) is rare; its occurrence in the gastrointestinal tract is of exceedingly low prevalence. We report a case of a 73-year-old man with metastatic malignant melanoma to the colon from an unknown primary. The rarity of MUP and importance of screening for gastrointestinal metastasis in patients with malignant melanoma are discussed along with the role of surgical resection in improving prognosis and overall survival.

## 1. Introduction

Approximately 1–3% of all malignant tumors of the gastrointestinal tract (GIT) are secondary to metastatic malignant melanoma [1]. Only 1–4% of all melanoma cases per year are from an unknown primary, while fewer than 7% of these cases are found in the colon [2–5]. We present a case of malignant melanoma with an unknown primary (MUP) metastasizing to the colon. The rarity of metastatic malignant melanoma of the colon, particularly from an unknown primary site, is discussed along with the importance of surgical resection in improving prognosis and overall survival.

## 2. Case

A 73-year-old Caucasian man presented with an enlarging, tender, 8 mm subcutaneous mass in his left cheek. He has a past medical history of Parkinson's disease and monoclonal gammopathy of undetermined significance (MGUS). He denied prior history of skin cancer, excised melanotic nevi, other skin and ocular lesions, and family history of melanoma. Physical examination revealed a firm, immobile mass involving his left cheek without evidence of cervical, axillary, and groin lymphadenopathy or skin lesions with atypical features. Computed tomography (CT) of the head with and without contrast revealed a round, nonenhancing soft tissue density overlying the left cheek anterior to the left masseter muscle; fine needle aspiration of the mass demonstrated malignant melanoma with immunohistochemical studies positive for melan-A and HMB-45. A complete positive electron tomography (PET) was negative for distant metastasis.

He underwent a left total parotidectomy and cervical lymph node dissection for metastatic malignant melanoma of the parotid gland with an unknown primary site. Three out of the twelve cervical lymph nodes taken from the neck dissection were positive for melanoma, one of which had extra-nodal extension. Gross residual disease was treated with radiation therapy. The patient declined adjuvant therapy with interferon due to the neuropsychiatric effects it may have on his Parkinson's disease. Two months later, the patient required transoral resection of the left buccal pad due to recurrence of tumor in the left buccal space, followed by radiation therapy to the area.

**Figure 1:**
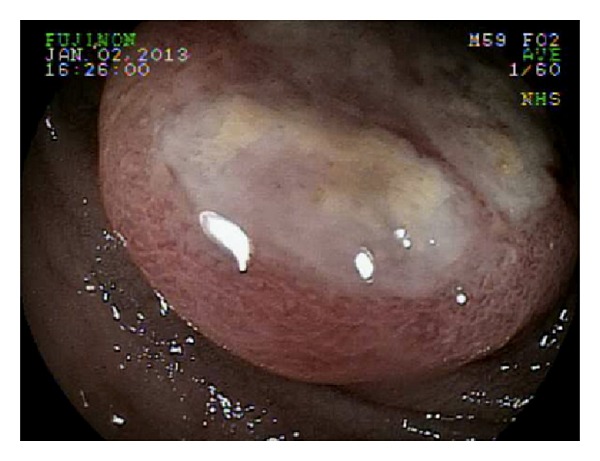
Colonoscopy showing a 4 × 4 cm villous, fungating, nonobstructing, noncircumferential, and nonbleeding mass in the sigmoid colon.

**Figure 2:**
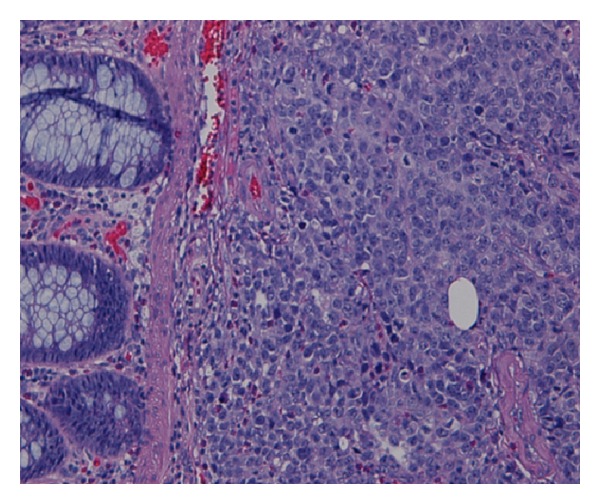
Sigmoid colon mass biopsy showing neoplastic proliferation of cells within the mucosal layer.

**Figure 3:**
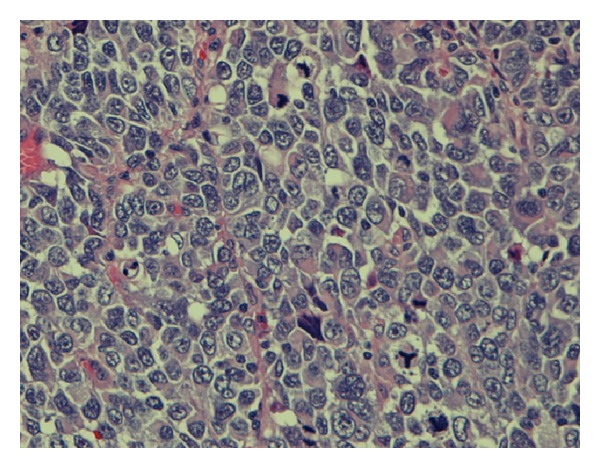
High magnification of the tissue biopsy showing irregular nuclear borders, condensed chromatin, and numerous atypical mitoses.

**Figure 4:**
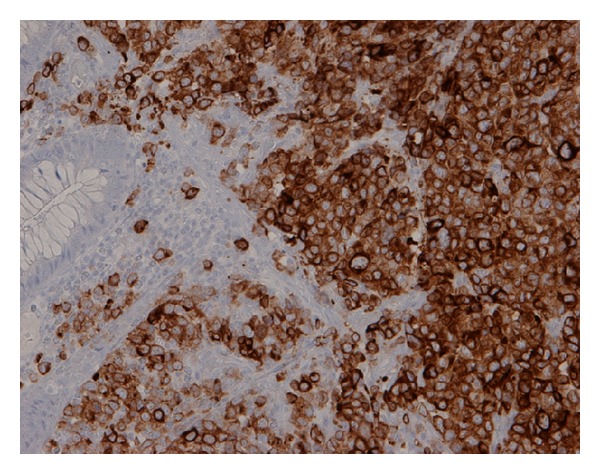
Immunohistochemistry of biopsy tissue showing melan-A positivity.

Seven months after recurrence, a follow-up PET scan showed a sigmoid colon lesion and a right adrenal nodule. He was asymptomatic. Abdominal and rectal exams were unrevealing. Complete blood count was unremarkable. Biochemistry studies including liver function tests and lactate dehydrogenase were within normal limits. Colonoscopy revealed a nonbleeding, villous, fungating, nonobstructing, and noncircumferential mass approximately 4 cm in length and diameter in the sigmoid colon (Figure 1). Histology of the mass showed neoplastic proliferation of the cells within the mucosal layer (Figure 2). Higher magnification showed irregular nuclear borders, condensed chromatin, and numerous atypical mitoses (Figure 3). Immunohistochemistry was positive for melan-A (Figure 4). All of these findings are consistent with malignant melanoma. The patient subsequently underwent a combined laparoscopic resection of his sigmoid colon and right adrenalectomy with surgical pathology confirming metastatic melanoma to the sigmoid colon.

## 3. Discussion

Melanomas have the potential to evolve from any site of the body containing melanocytes or cells that are capable of differentiating into melanocytes, which can be either cutaneous or noncutaneous in origin [6]. Malignant melanoma to the colon rarely occurs, however, due to the absence of melanocytes in the colon [7].

Melanoma without an identifiable cutaneous, ocular, or mucosal primary comprises only 1–4% of melanoma cases per year [2, 3]. Giuliano et al. reported a study of 980 cases of MUP, less than 7% of which had metastasis to the colon [2]. In multiple case series, the lymph nodes were found to be the most common site of metastasis for MUP. Various ideas have been postulated regarding the etiology of MUP. The model of spontaneous regression is the most widely accepted theory as it has been found to be especially applicable to melanoma [8]. Spontaneous regression may be due to alterations in immunologic status and can be triggered by stresses to the body including infection and pregnancy [6]. An unrecognized or missed primary on physical exam, misdiagnosis of a previously excised tumor on histology, and malignant transformation of ectopic nevus cells are among other explanations [2, 4, 5, 9].

The sigmoid colon mass found in this case is thought to be secondary to metastatic melanoma to the GI tract rather than a primary mucosal melanoma. The presence of primary melanoma of the colon, or in any other portion of the GI tract aside from the esophagus and anorectum, where melanocytes normally exist, has been questionable. Sachs et al. proposed that primary intestinal melanoma (1) is a solitary lesion, (2) has no evidence of metastasis to other organs, (3) has a precursor lesion or histological evidence of melanosis, and (4) has a disease-free interval (DFI) of at least 12 months after diagnosis [10]. The patient had no findings suggestive of a precursor lesion in the colon and had adrenal metastasis. These findings suggest metastatic melanoma of the colon. What initially distinguished the colon mass as metastatic melanoma versus a primary mucosal melanoma was that this patient had known metastatic melanoma of the parotid gland. Although extremely rare, comprising only 0.68% of all malignancies found in the parotid gland, the possibility of a primary parotid melanoma was considered and cannot be absolutely ruled out [11, 12].

Many malignant tumors are amelanotic and have atypical histology making the diagnosis of melanoma difficult. Most melanomas stain positive for S-100 and HMB-45 [13]. Our patient's sigmoid colon mass was amelanotic but stained positive for melan-A, also commonly found in metastatic melanomas. S-100 is highly sensitive while HMB-45 and melan-A are highly specific in diagnosing malignant melanoma [6].

Most patients will not present with such symptoms as abdominal pain, anemia, abdominal obstruction, or tenesmus to suggest gastrointestinal involvement. Only 1 to 4% of all patients with malignant melanoma have clinically apparent GIT involvement and are diagnosed antemortem, while up to 60% of all patients with melanoma are found to have metastasis at autopsy [14–17]. Given these findings, patients with known melanoma should be screened for gastrointestinal spread. Currently, dual modality PET-CT is the most accurate in detecting and staging melanoma metastases [18, 19]. Colonoscopy has the greatest diagnostic value with high sensitivity and specificity and allows for tissue biopsy.

Patients with limited disease tend to have better survival following complete tumor resection. A study by Akaraviputh et al. at a surgical training center found that patients who underwent curative resection had a longer mean survival time (29.7 months) [14]. Similarly, a retrospective study by Panagiotou et al. showed that those with malignant melanoma to the GI tract had a median survival of 46.7 months following curative surgical resection, while those treated nonsurgically had a median survival of 5.8 months [15]. Alternative therapies to surgical resection of colon melanoma metastases include chemotherapy, biochemotherapy, and radiation. In a retrospective study of 10,365 patients, Mourra et al. found that surgery with complementary therapy can prolong survival in some patients [20]. Adjuvant therapy, including radiation, chemotherapy, and immunotherapy, is utilized for residual disease or nodal metastasis following surgical resection. However, its effectiveness continues to be controversial. Our patient underwent radiation to residual disease in the parotid bed and the involved lymph node that had extranodal extension, following parotidectomy. However, his disease recurred 2 months later. Perhaps he failed radiation therapy because the parotid melanoma was not excised to achieve negative margins or due to the lack of therapy with interferon.

The prognosis and overall survival of patients with MUP in other previously reported series have been similar to those of patients with a known primary in the same stage [2]. A cohort study done by Katz et al. at Massachusetts General Hospital and Dana-Farber Cancer Institute showed that there was no difference in the 5-year survival rates of patients with MUP and the controls with known primaries [3]. Further, Giuliano et al. found that the overall recurrence rate did not differ among patients with MUP and those with a known primary [2]. Colon metastasis indicates late stage disease with a poor prognosis. Surgical resection has been shown to improve prognosis by 23–48 months in patients with metastatic malignant melanoma of the GI tract [21]. In fact, Wysocki et al. presented a case of patient who achieved long-term disease-free survival of 101 months after surgical resection of metastatic malignant melanoma to ileum and colon [22]. Postoperatively, our patient had an uncomplicated course, and 3 months later, routine imaging and physical examination show that he is disease-free and still without evidence of a primary site, respectively. He continues to undergo long-term followup, as recurrence of disease is likely.

Differentiating primary malignant melanoma from metastatic malignant melanoma, if possible, can be very challenging. The idea of metastasis from an unknown primary, possibly from tumor regression, must be entertained especially if the initial lesion would rarely present as a primary malignancy. Obtaining a thorough history with questions honing in on prior excised lesions, progressive skin lesions, prior skin and ocular lesions, and personal and family history of melanoma is crucial. Thorough dermatological and ocular exams are particularly important in looking for a primary. It is important for patients with melanoma, even after treatment, to undergo routine imaging not only to look for recurrence of disease but also to rule out GIT metastasis as it rarely manifests clinically. Early diagnosis is the key in initiating surgical intervention asresection not only is palliative but also can improve both prognosis and overall survival, including long-term, disease-free survival.

## References

[1] Blecker D, Abraham S, Furth EE, Kochman ML (1999). Melanoma in the gastrointestinal tract. *The American Journal of Gastroenterology*.

[2] Giuliano AE, Moseley HS, Morton DL (1980). Clinical aspects of unknown primary melanoma. *Annals of Surgery*.

[3] Katz KA, Jonasch E, Hodi FS (2005). Melanoma of unknown primary: experience at Massachusetts General Hospital and Dana-Farber Cancer Institute. *Melanoma Research*.

[4] Anbari KK, Schuchter LM, Bucky LP (1997). Melanoma of unknown primary site: presentation, treatment, and prognosis—a single institution study: University of Pennsylvania Pigmented Lesion Study Group. *Cancer*.

[5] Vijuk G, Coates AS (1998). Survival of patients with visceral metastatic melanoma from an occult primary lesion: a retrospective matched cohort study. *Annals of Oncology*.

[6] Khalid U, Saleem T, Imam AM, Khan MR (2011). Pathogenesis, diagnosis and management of primary melanoma of the colon. *World Journal of Surgical Oncology*.

[7] Schuchter LM, Green R, Fraker D (2000). Primary and metastatic diseases in malignant melanoma of the gastrointestinal tract. *Current Opinion in Oncology*.

[8] McGovern VJ (1975). Spontaneous regression of melanoma. *Pathology*.

[9] Krishna Mohan MV, Rajappa SJ, Reddy TV, Paul TR (2009). Malignant gastrointestinal melanoma with an unknown primary. *Indian Journal of Medical and Paediatric Oncology*.

[10] Sachs DL, Lowe L, Chang AE, Carson E, Johnson TM (1999). Do primary small intestinal melanomas exist? Report of a case. *Journal of the American Academy of Dermatology*.

[11] Tsutsumida A, Yamamoto Y, Sekido M, Itoh T (2008). Suspected case of primary malignant melanoma of the parotid gland. *Scandinavian Journal of Plastic and Reconstructive Surgery and Hand Surgery*.

[12] Bahar M, Anavi Y, Abraham A, Ben-Bassat M (1990). Primary malignant melanoma in the parotid gland. *Oral Surgery Oral Medicine and Oral Pathology*.

[13] Prayson R, Sebek BA (2000). Parotid gland malignant melanomas. *Archives of Pathology and Laboratory Medicine*.

[14] Akaraviputh T, Arunakul S, Lohsiriwat V, Iramaneerat C, Trakarnsanga A (2010). Surgery for gastrointestinal malignant melanoma: experience from surgical training center. *World Journal of Gastroenterology*.

[15] Panagiotou I, Brountzos EN, Bafaloukos D, Stoupis C, Brestas P, Kelekis DA (2002). Malignant melanoma metastatic to the gastrointestinal tract. *Melanoma Research*.

[16] Serin G, Doğanavşargil B, Çalişkan C, Akalin T, Sezak M, Tunçyürek M (2010). Colonic malignant melanoma, primary or metastatic? Case report. *Turkish Journal of Gastroenterology*.

[17] Washington K, McDonagh D (1995). Secondary tumors of the gastrointestinal tract: surgical pathologic findings and comparison with autopsy survey. *Modern Pathology*.

[18] Reinhardt MJ, Joe AY, Jaeger U (2006). Diagnostic performance of whole body dual modality 18F-FDG PET/CT imaging for N- and M-staging of malignant melanoma: experience with 250 consecutive patients. *Journal of Clinical Oncology*.

[19] Holder WD, White RL, Zuger JH, Easton EJ, Greene FL (1998). Effectiveness of positron emission tomography for the detection of melanoma metastases. *Annals of Surgery*.

[20] Mourra N, Jouret-Mourin A, Lazure T (2012). Metastatic tumors to the colon and rectum, a multi-institutional study. *Archives of Pathology and Laboratory Medicine*.

[21] Wook K, Jong MB, Young JS, Jeon HM, Kim JA (2004). Ileal malignant melanoma presenting as a mass with aneurysmal dilatation: a case report. *Journal of Korean Medical Science*.

[22] Wysocki WM, Komorowski AL, Darasz Z (2004). Gastrointestinal metastases from malignant melanoma: report of a case. *Surgery Today*.

